# Evaluating real-time immunohistochemistry on multiple tissue samples, multiple targets and multiple antibody labeling methods

**DOI:** 10.1186/1756-0500-6-542

**Published:** 2013-12-18

**Authors:** Louise Dubois, Karl Andersson, Anna Asplund, Hanna Björkelund

**Affiliations:** 1 Department of Radiology, Oncology, and Radiation Sciences; Biomedical Radiation Sciences, Rudbeck Laboratory, Uppsala University, 75185 Uppsala, Sweden; 2 Ridgeview Instruments AB, Skillsta 4, 74020 Vänge, Sweden; 3 Ridgeview Diagnostics AB, Uppsala Science Park, 75183 Uppsala, Sweden; 4 Department of Immunology, Genetics and Pathology and Science for Life laboratory, Rudbeck Laboratory, Uppsala University, 75185 Uppsala, Sweden

**Keywords:** Antibodies, HER2, IHC, Kinetics, LigandTracer, RBM3, Real-time, SATB2

## Abstract

**Background:**

Immunohistochemistry (IHC) is a well-established method for the analysis of protein expression in tissue specimens and constitutes one of the most common methods performed in pathology laboratories worldwide. However, IHC is a multi-layered method based on subjective estimations and differences in staining and interpretation has been observed between facilities, suggesting that the analysis of proteins on tissue would benefit from protocol optimization and standardization. Here we describe how the emerging and operator independent tool of real-time immunohistochemistry (RT-IHC) reveals a time resolved description of antibody interacting with target protein in formalin fixed paraffin embedded tissue. The aim was to understand the technical aspects of RT-IHC, regarding generalization of the concept and to what extent it can be considered a quantitative method.

**Results:**

Three different antibodies labeled with fluorescent or radioactive labels were applied on nine different tissue samples from either human or mouse, and the results for all RT-IHC analyses distinctly show that the method is generally applicable. The collected binding curves showed that the majority of the antibody-antigen interactions did not reach equilibrium within 3 hours, suggesting that standardized protocols for immunohistochemistry are sometimes inadequately optimized. The impact of tissue size and thickness as well as the position of the section on the glass petri dish was assessed in order for practical details to be further elucidated for this emerging technique. Size and location was found to affect signal magnitude to a larger extent than thickness, but the signal from all measurements were still sufficient to trace the curvature. The curvature, representing the kinetics of the interaction, was independent of thickness, size and position and may be a promising parameter for the evaluation of e.g. biopsy sections of different sizes.

**Conclusions:**

It was found that RT-IHC can be used for the evaluation of a number of different antibodies and tissue types, rendering it a general method. We believe that by following interactions over time during the development of conventional IHC assays, it becomes possible to better understand the different processes applied in conventional IHC, leading to optimized assay protocols with improved sensitivity.

## Background

Immunohistochemistry (IHC) is a widely used technique for diagnostic and research purposes [1]. Its central role in classification of diseases by evaluation of receptors and other cellular components in biopsies and surgical resections has made IHC one of the most important methods in pathology [2].

The basics of IHC are to stain a thin representative tissue section and evaluate the intensity and localization of the staining in order to understand e.g. antigen expression. Estimation of the level and distribution of expression is subjectively performed by trained personnel through visual inspection in the microscope, and commonly staining positivity is described as -, +, ++ and +++. The technique provides superior spatial resolution, but is operator dependent and further relies on multi-layered end-point measurements which increase the risk of inaccurate report of antigen expression. One example is the reporting of HER2 expression in bladder cancer (2-79%) [3,4] and ovarian cancer (5-100%) [5,6]. The problems associated with IHC have been discussed more intensively the last couple of years and there is a general call for standardization [7,8].

Recently, Gedda et al. reported a method allowing the time resolved measurement of the interaction between a radiolabeled antibody and its antigen as expressed in tissue – real-time IHC (RT-IHC) [9]. The use of real-time measurements compared to manual end-point measurements has many benefits, such as an inherent and immediate quality control where problems with an assay can be detected as e.g. irregularities in the binding curve. The time-resolution opens up for estimations of time to equilibrium, reducing the risk of interrupting the incubation prematurely. Thus, time resolved detection technologies are particularly useful when developing novel assays and when troubleshooting existing assays. The possibility to follow the interaction minute by minute can not only reveal when equilibrium occurs, but may also pinpoint which of several components in a multilayer technique that is the limiting factor [10]. It has further been demonstrated that the curvature of the real-time binding trace, corresponding to the kinetics of the antibody-antigen interaction, contain important information about the tissue, where e.g. non-specific binding can be corrected for based on its distinct time-resolved binding pattern. For example, in a second study by Gedda et. al., a clear correlation was found between the curve shape and the HER2 scoring of biopsy material from twenty breast cancer patients [11], independent of signal amplitude.

All measurements conducted in this study were done using LigandTracer® instruments. LigandTracer was initially developed for monitoring protein-cell interactions in real time [12-16], but is equally applicable to evaluate interactions to paraffin embedded tissue [9] or between purified proteins [17]. Although the applications vary, the basic principle of the instrument is the same. A target area of a Petri dish is created by e.g. mounting tissue sections or seeding adherent cells. The dish is placed on a tilted, rotating support and a solution with a fluorescently or radioactively labeled molecule is added. The signal detection is performed in the elevated area of the dish and followed over time. A target free area of the dish is used as a reference to continuously subtract the background signal.

In this work three different antigens were analyzed: SATB2, RBM3 and HER2. Low expression of the cold shock RNA-binding protein RBM3 is associated with tumor progression and poor prognosis in malignant melanoma [18]. The DNA-binding SATB2 protein is known to be involved in transcriptional regulation and chromatin remodeling [19] and mutations of its corresponding gene has been correlated with cleft palate and cognitive defects [20]. The HER2 membrane receptor is a member of the epidermal growth factor receptor family and is overexpressed in e.g. breast cancer [21], making it an important target for anti-cancer therapies such as the therapeutic antibody Herceptin® [22].

The previous work with RT-IHC has been done with one single antibody, one radioactive label and mostly mouse tissue or human xenograft in mouse. This work aimed at investigating if the method was generally applicable by the use of different antigens, antibodies, labels (including two fluorophores) and tissue types. The setup of the two previous studies was also included [9,11], as a link between old and new data. All other tissues were chosen to represent strong and weak staining as explored using conventional IHC. Use of fluorescent labels is essential for making the assay accessible for all laboratories, as radioactivity requires proper facilities and special type of instruments. This paper further evaluates how tissue size, thickness and dish position can affect RT-IHC results.

## Results

### IHC analysis

**Figure 1:**
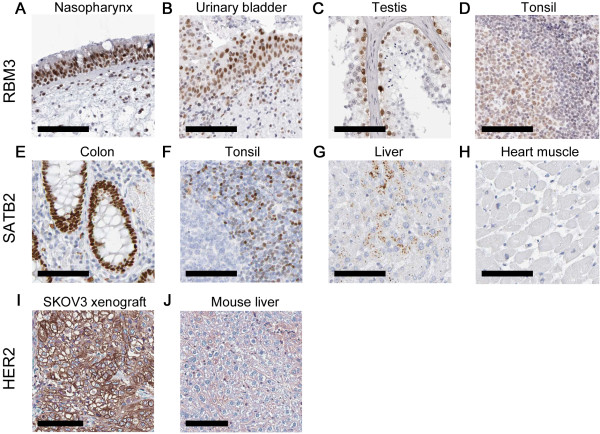
**Tissue staining of RBM3, SATB2 and HER2*.*** Staining of RBM3 in **A)** nasopharynx, **B)** urinary bladder, **C)** testis, **D)** tonsil, SATB2 in **E)** colon, **F)** tonsil, **G)** liver, **H)** heart muscle, and HER2 in **I)** SKOV3 xenograft, **J)** mouse liver. The primary antibody was incubated for 30 minutes in all stainings, according to the established protocol. The scale bar indicates 100 μm.

Tissues were selected to represent a range of staining intensities for the anti-RBM3 and anti-SATB2 antibodies (Figure 1). For the antibody binding to RBM3, nasopharynx and urinary bladder were selected for representing strong staining, testis for representing strong staining in only on a subset of the cells in the tissue, and tonsil for signs of only weak staining. For the anti-SATB2 antibody, colon displayed strong staining, tonsil and liver heterogenous staining with scattered areas of positivity and heart muscle was negative. The tissues used as positive and negative control for HER2 in previous studies were included as well [9,11]. The anti-HER2 antibody stained the SKOV3 xenograft strongly and mouse liver tissue weakly.

### The impact of different incubation times

**Figure 2:**
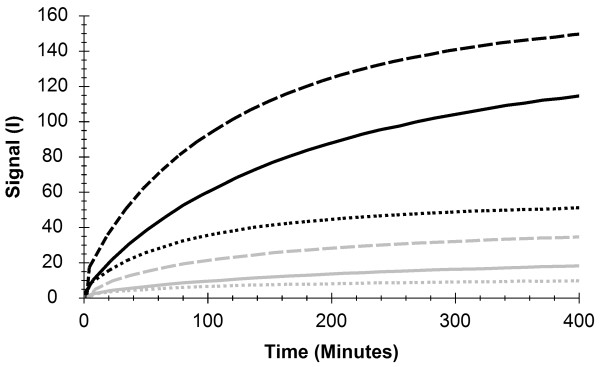
**RT-IHC results showing RBM3 expression using fluorescently labeled secondary antibody*.*** The binding of secondary antibody in nasopharynx (black lines) and tonsil (grey lines) after 1 h (dotted lines), 3 h (dashed lines) and 24 h (solid lines) of pre-incubation with a RBM3-binding antibody. I = Fluorescent intensity units.

The effect of varying primary antibody pre-incubation times (1 h, 3 h or 24 h) prior to measurement of labeled secondary antibody was conducted using 8 nM unlabeled anti-RBM3 antibody, with the RBM3 high expressing nasopharynx and the RBM3 low expressing tonsil (Figure 2). Clear binding traces were collected in the instrument LigandTracer Green using 6.5 nM fluorescently labeled secondary antibody. One hour pre-incubation with 8 nM anti-RBM3 antibody produced the lowest signals, suggesting that more than one hour is beneficial to obtain high signal levels. Irrespective of pre-incubation time, the signal from tonsil was small in comparison to the signal from nasopharynx, which is in line with the results from IHC analysis presented in Figure 1. The secondary antibody required more than 300 minutes to reach equilibrium at the used concentration (6.5 nM).

### Comparison of labeled primary and secondary antibody in RT-IHC

**Figure 3:**
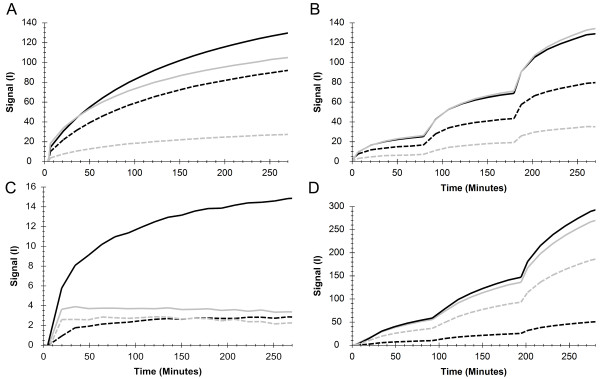
**RT-IHC results, using fluorescently labeled primary or secondary antibody*.*** Binding traces produced in LigandTracer, depicting the binding of **A)** Alexa Fluor 488 labeled anti-mouse antibody after 3 h pre-incubation with anti-RBM3 antibody, **B)** direct binding of Texas Red-labeled anti-RBM3 antibody at increasing concentrations, added stepwise (6, 12, 21 nM – 80-100 min incubation of each concentration), **C)** Alexa Fluor 488 labeled anti-mouse antibody after 3 h pre-incubation with anti-SATB2 antibody, **D)** direct binding of Texas Red-labeled anti-SATB2 antibody at increasing concentrations, added stepwise (6, 12, 21 nM – 80-100 min incubation of each concentration). In A and B, measurements were done in nasopharynx (black solid line), urinary bladder (black dashed line), testis (grey solid line) and tonsil (grey dashed line). Measurements C and D were performed in colon (black solid line), tonsil (black dashed line), liver (grey solid line) and heart muscle (grey dashed line). I = Fluorescent intensity units.

A panel of tissues was used for comparing the real-time binding of 6.5 nM fluorescently labeled secondary antibody to tissues pre-incubated (3 h) with 8 nM unlabeled anti-RBM3 antibody (Figure 3A). Nasopharynx yielded the highest signal and tonsil the lowest. For urinary bladder and testis, the signals were slightly lower than for nasopharynx, which is in agreement with the tissue staining. The results were similar when monitoring the binding of labeled primary antibody (anti-RBM3) at increasing concentrations (6, 12, 21 nM), although with a somewhat higher signal for testis (Figure 3B). The time for 6 nM of primary antibody to reach equilibrium was approximately 100 minutes, as estimated from Figure 3B.

A similar pair of experiments was conducted towards SATB2 (Figure 3C, D). In both cases, colon yielded the highest signal and tonsil the lowest. The secondary antibody produced low signals for heart muscle and liver (Figure 3C), but the signal level of liver was comparable to that of colon during the detection of labeled primary antibody (Figure 3D). Time for 6 nM of the anti-SATB2 antibody to reach equilibrium was greater than 100 minutes (Figure 3D).

### The binding of anti-HER2 antibody to SKOV3 xenograft and mouse liver

**Figure 4:**
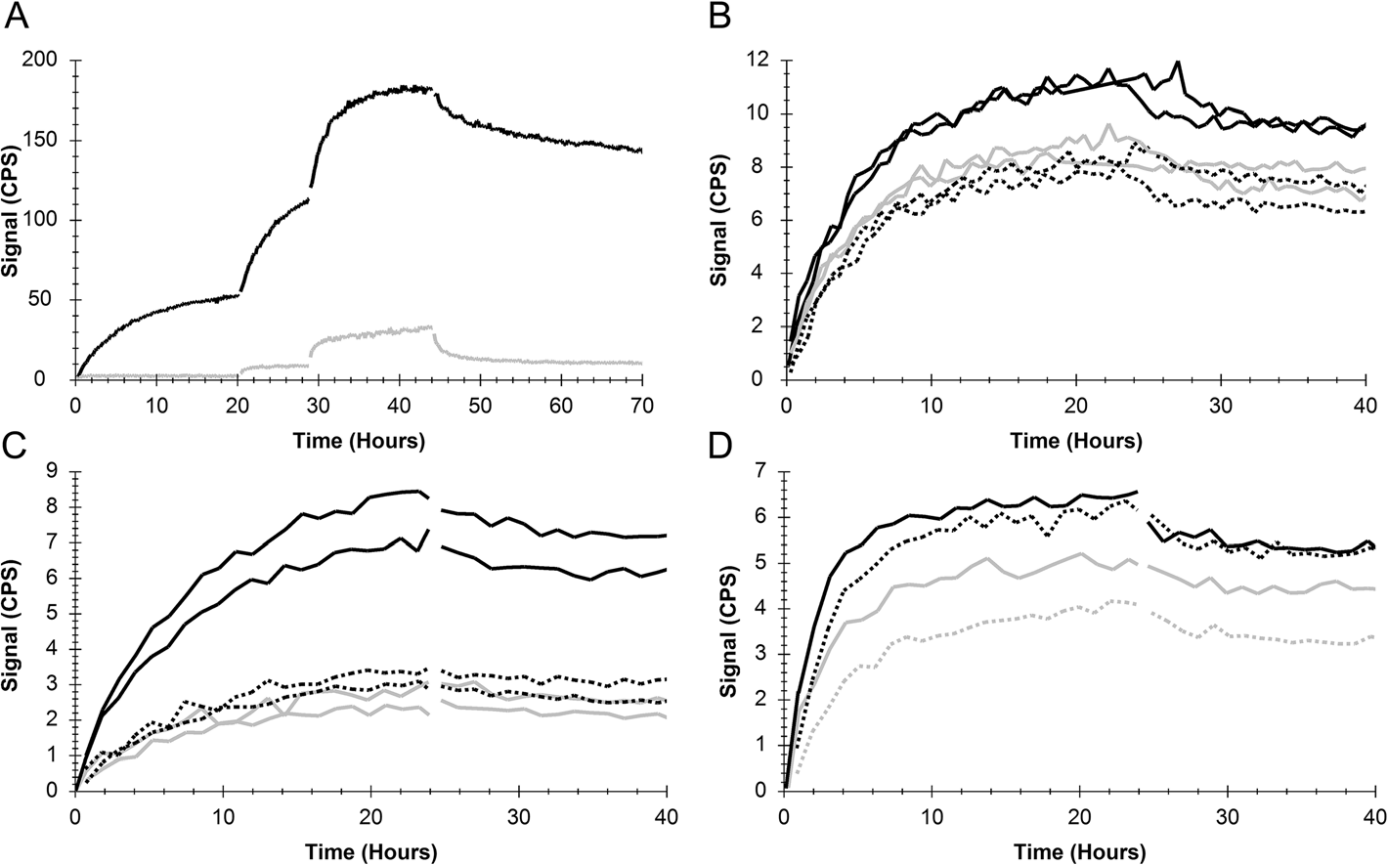
**The impact of tissue thickness, size and position on RT-IHC data*. *A)** 1, 3 and 9 nM ^125^I-labeled anti-HER2 antibody subsequently added in the same measurement at t = 0 h (1 nM), t = 20 h (3 nM) and t = 29 h (9 nM) to a dish containing SKOV3 xenograft (black) and mouse liver (grey), with high and low HER2 expression respectively. The antibody solution was replaced with PBS + 1% BSA (t = 44 h) in order to study the dissociation rate. **B)** 1 nM ^125^I-antibody binding to 10 μm (black solid line), 4 μm (black dotted line) and 2 μm (grey solid line) thick SKOV3 xenograft tissue slices. **C)** 1 nM ^125^I-antibody binding to SKOV3 xenograft tissues of different sizes: A full size tissue slice (black solid line), half a slice (black dotted line) or a quarter of a slice (grey solid line). **D)** 1 nM ^125^I-antibody binding to SKOV3 xenografts positioned approximately 0-16 mm (black solid line), 4-20 mm (black dotted line), 8-24 mm (grey solid line) or 12-28 mm (grey dotted line) from the rim of the dish. The detection area stretches from the rim and into approximately 20 mm of the dish. CPS = Counts per second.

The concentration of ^125^I-labeled anti-HER2 antibody was step-wised increased (1 nM, 3 nM, 9 nM; 10-20 h per concentration) in a dish containing HER2 high expressing SKOV3 xenograft and HER2 low expressing mouse liver, followed by a dissociation phase (0 nM). A clear interaction was seen to the SKOV3 xenograft (black curve), but to a much lower extent to the mouse liver tissue. The interaction was slow, requiring approximately 24 h to reach equilibrium for the lowest concentration (Figure 4A), which is in line with what has previously been observed [9]. The significant signal increase upon addition of antibody suggests that the HER2 on the SKOV3 xenograft is far from saturated for the lower concentrations.

### Studying the impact of tissue thickness, size and position

The binding of anti-HER2 antibody with the SKOV3 xenograft was detected in various conditions in order to study the impact of tissue thickness, size and position.

The thickness of the tissue sections appears to have a relatively small impact on the signal level in RT-IHC. 2 μm and 4 μm thicknesses resulted in almost identical signal levels, while the 10 μm slices produced approximately 35% higher signals (Figure 4B). The different tissue thicknesses were measured in parallel in one dish, at two occasions (generating duplicates of each thickness type).

The tissue slices were cut into different sizes (full size, half a slice and a quarter of a slice) and measured in parallel in the same dish at two occasions. A correlation between signal level and tissue size was observed (Figure 4C). The full sized slice (black solid line) produced approximately twice as high signal than the half slice (black dotted line). However, the signal level from the half slice and the quarter slice (grey solid line) were similar.

Positioning of the tissue section is relevant if this causes some part of the tissue slice to be outside the detection area, which stretches from the rim and into approximately 20 mm of the dish (Figure 4D) for LigandTracer Grey. With 17 mm in length, the slices were completely or almost completely within the detection area when positioned 0 mm (black solid line) or 4 mm (black dotted line) from the rim of the dish. This resulted in somewhat higher signal levels for the 0 mm position. When positioned 8 mm (grey solid line) or 12 mm (grey dotted line) a significant proportion of the tissue slices were undetected, producing clearly lower signal heights.

Tissue thickness, size and position did not affect the shape of the real-time binding curves, as observed after data normalization (data not shown).

## Discussion

Previous work on RT-IHC has been done with a single antibody, through detection of radioactivity [9,11]. This report summarizes results from several different real-time IHC assays, using a series of tissues, labels and use of primary labeled antibodies or secondary antibody readout. Thus, we have verified the work of Gedda et. al. and showed that real-time IHC is generally applicable in histology, also for fluorescently labeled antibodies. Another aim of this study was to investigate which conditions of the tissue (thickness, size and position) that can have an impact on the result and within which limits sufficiently high signal levels are produced.

All antibody-antigen interactions investigated in this study, both the primary antibody binding to its target and the secondary antibody binding to the primary, require several hours to reach equilibrium at the concentrations used. This is in line with previous reports from both general antibody binding and real-time IHC [9,23,24]. Common IHC protocols typically rely on short incubation times (about 30 minutes) and low concentrations of antibody, meaning that the likelihood that equilibrium is approached during incubation is low. This is probably one complicating factor in the reproducibility of IHC because if the assay is performed without reaching equilibrium, small variations in incubation time and antibody concentration may change the binding level of the antibody and thereby alter the end result. It has further been observed that the binding rate of the same antibody can vary between tissues, which will result in different staining intensities if equilibrium is not reached, even if the antigen is expressed to the same degree [11].

The results from the tissue staining were similar to the results of the secondary antibody measured in LigandTracer (Figure 3A, C). The low signal levels for tonsil and liver pre-incubated with anti-SATB2 antibody (Figure 3C) is likely the result of SATB2 being expressed in only a small fraction of the tissue (Figure 1F, G).

There are differences between the results of the primary (Figure 3B, D) and secondary antibody (Figure 3A, C) measurements. In the tissue panel used for RBM3, testis had higher signals from primary labeled antibody than from secondary read out (in comparison to other tissues), and the same deviation was observed for liver and heart muscle for SATB2. At this point we can only observe that there is a discrepancy, finding out the origin of it is beyond the scope of this paper. One possible reason is that the deviating tissues (testis, liver and heart muscle) have low-affinity sites to which the primary antibody binds, but also dissociates quickly after wash. Such interactions with fast off-rate would remain undetected when using labeled secondary antibodies. These kind of weak interactions have been noted in the field of immunogenicity of therapeutic antibodies and proved critical for understanding the biological action [25].

The HER2 binding antibody was included in this study and labeled with ^125^I to facilitate comparison with previous work with RT-IHC [9,11]. Since both binding rate and signal height is comparable with the published data when using the same tissue types, the authors conclude that their RT-IHC protocol is similar to the earlier setup. Based on the results in Figures 2 and 3, a fluorescent label would have worked just as well.

The impact of thickness, size and position were evaluated in order to find optimal settings for the assay, as well as to investigate how small differences between tissue sections affect the signal height, curvature (interaction kinetics) and overall interpretation of RT-IHC data.

It was found that tissue thickness in some cases affected the signal level, as observed for the 10 μm sections. This is in line with what have been previously observed for manual scoring [26]. Fortunately, this is a property that can be controlled during processing, minimizing the risk of unwanted variation between samples due to thickness. The authors suggest the use of 3 - 4 μm sections since this is a commonly used thickness which also avoids waste of e.g. limited patient material. If a binding would be difficult to detect due to e.g. low expression levels, thicker sections can be used instead. Tissue size has a clear impact on signal height, but the relationship is not completely linear. The reason behind the similar signal levels for the half and quarter sized sections may be a heterogeneous distribution of the antigen (HER2), i.e. if the total amount of antigen is similar even though the tissue sizes are different. The overall conclusion from the studies represented in Figure 4 is that the signal height can be affected by tissue parameters such as size and positioning. This must be taken into consideration if the aim of RT-IHC is to use it as a quantitative tool for the comparison of antigen expression, especially when using patient biopsy material where the size and shape can vary. The strength of RT-IHC is however likely not the objective analysis of signal level, but rather the comparison of curvature, which was not affected by the thickness, size or positioning. For example, published data show that there is a correlation between curve shape, corresponding to interaction kinetics, and HER2 scoring [11]. A kinetic evaluation is independent of signal amplitude, as long as the signal is sufficient to trace the curvature. In this study the signal was high enough for a clear binding trace, but for low antigen expression it may be important to increase the amplitude. Based on the results of Figure 4, this is possible by e.g. using larger tissue sections.

One field where RT-IHC may be useful is in the development of conventional IHC. The ability to follow molecular interactions minute by minute is a help when developing assays, fine-tuning performance and troubleshooting problems. One obvious drawback of the RT-IHC method is the lack of spatial resolution, since one average binding trace is collected for the complete tissue section. As evident from the IHC results (Figure 1), it is not always the average signal from the complete section that is of interest. Hence RT-IHC should be seen as a complement to conventional IHC, where one method provides the temporal dimension and the other the spatial dimension.

## Conclusions

Real-time immunohistochemistry is confirmed as a generic tool for the development of conventional IHC. We will continue to explore the possibilities of RT-IHC in combination with conventional IHC in our laboratory.

## Methods

### Tissue samples and reagents

Tissue samples of nasopharynx, urinary bladder, testis, tonsil, colon, liver and heart muscle were obtained under the ethical permit of 2005:338, 2007/159 and 02-577.

Xenografts of the human ovarian carcinoma cell line SKOV3 (HTB-77, ATCC, Rocksville, MD, USA) were used to represent high expression of HER2. The xenografts were formed by s.c. inoculation in the right posterior leg of BALB/c (nu/nu) mice (M&B, Ry, Denmark) injecting 10^7^ SKOV3 cells in 0.1 ml medium, which comply with current Swedish law and done with permission from the local committee of animal research ethics. Once the xenografts had grown to a size of approximately 1 cm in diameter, tumors were excised and fixated in 4% phosphate-buffered formalin. Mouse liver, used as a HER2 low expressing tissue, was removed from healthy animals and fixated. Tissue was dehydrated for 15 + 15 min in 70% EtOH, 30 min in 90% EtOH, 15 + 15 min in 95% EtOH, 15 + 15 min in 99% EtOH and thereafter embedded in paraffin and stored until further use.

Mouse monoclonal antibodies against RBM3 (Product ID: AMAb90655, Clone ID CL0296) and SATB (Product ID: AMAb90635, Clone ID CL0276) were obtained from Atlas Antibodies AB (Stockholm, Sweden). The Alexa Fluor 488 labeled goat anti-mouse IgG (Product ID: A11001) used as a secondary antibody was from Invitrogen (Carlsbad, California). The polyclonal antibody directed against HER2 (Product ID: A0485) was obtained from DAKO (Glostrup, Denmark). Although being of polyclonal nature, the authors have previously demonstrated that this antibody has a highly monoclonal interaction behavior, i.e. one single dominating interaction with defined kinetic properties was observed when binding to the SKOV3 xenograft [11].

### Antibody labeling

Primary antibodies for SATB2 and RBM3 were labeled with Texas Red to the lysine residues according to the manufacturer’s instructions. The fluorophore was dissolved in DMSO and mixed with 10 μg of antibody in a total volume of approximately 100 μl borat buffer pH 9. After 60-90 minutes of incubation at 37°C the unreacted dye was separated from the labeled antibody using a NAP-5 column. The labeled antibodies were wrapped in tin foil and stored in the freezer. The secondary antibody (anti-mouse) was labeled with Alexa Fluor 488 by the manufacturer.

The anti-HER2 antibody was labeled with ^125^I according to the CAT-protocol [27], which causes ^125^I to bind to tyrosine residues on the antibody.

### IHC analysis

Tissue sections (4 μm thick) were placed on FROST glass followed by heat incubation (50°C) over night. The sections were deparaffinized in a Leica (Wetzlar, Germany) autostainer and pressure boiled in a decloaking chamber from Biocare Medical (Concord, CA, USA) in TRS buffer pH 6. Finally, the tissue sections were manually scored, essentially as previously described [28]. In brief, tissue sections were incubated with primary antibody and a dextran polymer visualization system (UltraVision LP HRP polymer, Thermo Scientific, Waltham, MA, USA) for 30 min each at room temperature followed by incubation for 10 min with chromogen diaminobenzidine (Thermo Scientific, Waltham, MA, USA). All incubations were followed by a rinse in wash buffer (Thermo Scientific, Waltham, MA, USA). Slides were counterstained in Mayer’s hematoxylin (Histolab, Gothenburg, Sweden) for 5 min. No blocking step was included, which is in line with the findings of Buchwalow and colleagues [29].

### RT-IHC analysis

Multiple tissues sections were placed evenly distributed along the rim of a glass petri dish essentially as described previously [9]. One position was always left tissue free, to be used as a background reference.

Prior to measurement, dishes were deparaffinized in xylen 20 + 5 min, 99.5% EtOH 20 + 5 min, 95% EtOH 20 + 5 min, 70% 20 min and kept in dH2O until pressure boiled as described above. Dishes were left to cool off in running dH2O for a couple of minutes after pressure boiling. To avoid antigen degradation or tissue sections releasing from the glass dish, dishes were used the day of preparation.

The interaction of the antibody with its antigen was measured in LigandTracer Green or LigandTracer Grey (Ridgeview Instruments AB, Uppsala, Sweden), essentially as described by Björke [12], by monitoring either fluorescent (LigandTracer Green) or radioactive (LigandTracer Grey) labels. In brief, each dish was first rinsed gently with 4 ml PBS with 1% BSA for 15 minutes to coat all glass surfaces with protein, followed by mounting the dish in the instrument and adding 3 ml of fresh PBS with 1% BSA. After a short baseline measurement, labeled antibody was added and binding to the tissue sections were monitored during incubation. In some of the measurements (Figure 4), the incubation was followed by a dissociation measurement in which the antibody solution had been replaced with fresh PBS with 1% BSA. Corrections for nuclide decay were done automatically in the software controlling the instrument.

A few different antibody concentrations were tested for adequacy in RT-IHC. In the end, concentrations similar to the recommendations of the manufacturer (when available) were selected also for RT-IHC, since these resulted in detectable signals during LigandTracer measurements.

Interaction measurements of three types were conducted: (A) The tissue was pre-incubated with 8 nM unlabeled primary antibody followed by the detection of 6.5 nM labeled secondary antibody (SATB2, RBM3 – Figures 2 and 3A, C). (B) The tissue was used for direct detection of 6, 12 and 21 nM fluorescently labeled primary antibody, subsequently added (SATB2, RBM3 – Figure 3B, D). (C) The association and dissociation of antibody (1 nM or 1;3;9 nM) were monitored continuously (HER2 – Figure 4), using different tissue thicknesses (2 μm, 4 μm and 10 μm), sizes (100%, 50% and 25% the size of consecutive tissue slices) and positions (0 mm, 4 mm, 8 or 12 mm from the rim of the dish).

## Competing interests

K Andersson and H Björkelund are employed by and shareholders of Ridgeview Instruments AB.

## Authors’ contributions

LD carried out all of the laboratory work. KA conceived of the study and helped in the draft of the manuscript. AA evaluated the IHC data and helped in the draft of the manuscript. HB participated in the design and coordination of the study and performed all RT-IHC data evaluation and drafted the manuscript. All authors read and approved the final manuscript.
